# TNF-alpha expression, evaluation of collagen, and TUNEL of *Matricaria recutita L.* extract and triamcinolone on oral ulcer in diabetic rats

**DOI:** 10.1590/1678-775720150481

**Published:** 2016

**Authors:** Bruna Vasconcelos OLIVEIRA, Paulo Goberlânio BARROS SILVA, Jacqueline de Santiago NOJOSA, Luiz André Cavalcante BRIZENO, Jamile Magalhães FERREIRA, Fabrício Bitú SOUSA, Mário Rogério Lima MOTA, Ana Paula Negreiros Nunes ALVES

**Affiliations:** 1 - Universidade Federal do Ceará, Setor de Patologia Oral, Departamento de Odontologia Clínica, Fortaleza, CE, Brasil.; 2 - Universidade Federal do Ceará, Setor de Cariologia e Odontologia Restauradora, Departamento de Odontologia Restauradora, Fortaleza, CE, Brasil.; 3 - Universidade Estadual do Ceará, Setor de Fisiologia e Farmacologia, Departamento de Ciências Biomédicas, Fortaleza, CE, Brasil.; 4 - Universidade Federal do Ceará, Departamento de Fisiologia e Farmacologia, Fortaleza, CE, Brasil.

**Keywords:** Oral ulcer, Diabetes mellitus, Chamomile, Matricaria, Triamcinolone

## Abstract

Diabetes mellitus (DM) is a disease associated with delayed wound healing of oral ulcers by increased expression of proinflammatory cytokines and cellular apoptosis.

**Objective:**

to evaluate the influence of Tumor Necrosis Factor alpha (TNF-α) and apoptosis in rats with DM treated with chamomile extract or triamcinolone.

**Material and Methods:**

Wistar male rats (210.0±4.2 g) were divided into five groups: negative control group (NCG) without diabetes; positive control group (PCG) with DM (alloxan, 45 mg/kg); and groups treated with chamomile extract (normoglycemic= NCG group and diabetic= DCG group) and with triamcinolone (TG). Traumatic ulcers were performed on all animals that received topical triamcinolone, chamomile extract or saline 12/12 hours for ten days.

**Results:**

On days five and ten the animals were euthanized and the ulcers were analyzed by light microscopy, TUNEL assay, and immunohistochemically (TNF-α). The NCG (p=0.0062), PCG (p=0.0285), NCG (p=0.0041), and DCG (p<0.0001) groups were completely healed on the 10th day, however, there was no healing on the TG (p=0.5127) group. The TNF-α expression showed a significant reduction from the 5^th^ to the 10^th^ day in NCG (p=0.0266) and DCG (p=0.0062). In connective tissue, the TUNEL assay showed a significant reduction in the number of positive cells in NCG (p=0.0273) and CNG (p=0.0469) and in the epithelium only in CDG (p=0.0320).

**Conclusions:**

Chamomile extract can optimize the healing of traumatic oral ulcers in diabetic rats through the reduction of apoptosis in the epithelium and TNF-α expression.

## INTRODUCTION

Diabetes Mellitus (DM) is a chronic metabolic disease characterized by deficiency in insulin production or resistance to its action, resulting in hyperglycemia and metabolic alterations^4^. The incidence of DM is increasing in the world, and it is considered the biggest health problem in XXI century^18^. It is estimated that in 2025 there will be twice as many diabetic patients compared with the year 2000, totaling approximately 300 million affected individuals worldwide^20^.

Chronic hyperglycemia causes numerous events that promote structural changes in tissue. It is associated with delayed wound healing, increased susceptibility to infection, alterations in neutrophil activity, and reduction of chemotaxis, adhesion, phagocytosis and angiogenesis^4^ ^,^ ^7^. Clinically, the wound healing disorder manifests itself as hypertrophic scars or chronic unhealed wounds (ulcers), being ulcers the most prevalent problem in healing^18^.

In the oral cavity, traumatic ulcers are caused by mechanical trauma due to maladjusted dentures, orthodontic brackets, accidental bites, or iatrogenic factors. Typically, when the causal agent is removed, healing occurs spontaneously from one to two weeks; however, in a few cases, the ulcer can persist for longer periods of time. It can be extremely painful and interfere with eating and speaking^6^.

Corticosteroids are commonly prescribed for the treatment of painful symptoms of traumatic oral ulcers^6^; however, conflicting results have been reported in literature regarding the effects of this therapeutic modality on the healing process. Glucocorticoids have potent anti-inflammatory and immunosuppressive effects^1^. Triamcinolone is commonly used in clinical dentistry because of its analgesic effects on oral ulcers. It has a potent anti-inflammatory effect and is effective in reducing oral scores of mucositis and pain in patients undertaking radiotherapy^12^. But corticoids used in treatment of inflammatory conditions not only inhibit the symptoms of acute inflammation but also retard wound healing^1^ ^,^ ^12^.

In contrast, the use of natural products in the treatment of ulcerated oral lesions has increased over the past several decades^23^. The plant chamomile, also known as *Chamomilla recutita L.* or *Matricaria recutita L*., which is a member of the *Asteraceae* family, is one of the most widely used medicinal plants^10^. The fluid extract of chamomile has compounds, such as flavonoids (quercetin and apigenin), terpenes and acetylated derivatives, that confer anti-inflammatory effects, antibacterial, antifungal, antioxidant, hypocholesterolemic, and sedative properties^9^ ^,^ ^10^ ^,^ ^14^ ^,^ ^19^ ^,^ ^21^ ^,^ ^26^ ^,^ ^27^.

These drugs have been indiscriminately used in dental clinic for the treatment of persistent ulcerative lesions, but mechanisms of the diseases can be different in diabetic patients. Hyperglycemia lead keratinocytes and fibroblasts to high apoptosis levels that can modify the biological profile of wound closure and healing and interfere in collagen deposition^3^ ^,^ ^25^. Drugs used in the treatment of oral ulcer in diabetics should show a good efficacy in modifying these parameters. Thus, the aim of this study is to evaluate the influence of Tumor Necrosis Factor alpha (TNF-α) and apoptosis in rats with DM treated with chamomile extract or triamcinolone.

## MATERIAL AND METHODS

### Animals

This study was approved by the Ethics Committee for Animal Research (protocol no. 11/11), and was performed in accordance with the Ethical Principles in Animal Experimentation adopted by the Brazilian College of Animal Experimentation (COBEA).

We used male Wistar rats (*Rattus norvegicus albinus, Rodentia mammalia*) weighing 210.0±4.2 g (Mean±SD) that were provided by the Central Animal Facility of the Federal University of Ceará (UFC). The rats were kept in the Animal Laboratory of Experimental Oncology (LOE), given an initial examination for systemic health conditions and stored in boxes with sawdust. All animals received industrial feed (Bio-base^®^, Águas Frias, SC, Brazil) accordingly and water *ad libitum* and were kept at room temperature with controlled humidity for a photoperiod of 12 hours.

### Diabetes induction

The induction of diabetes was performed by injection of alloxan (45 mg/kg) in diluted sterile saline (0.9%) intravenously after mild sedation with ether. Two milliliters of blood were collected 48 h after the induction of diabetes from the retro-orbital plexus for determination of blood glucose. The animals were considered diabetic when the blood glucose was equal to or greater than 200 mg/dL^24^.

On the day of ulcer confection and sacrifice, blood was collected again for glucose measurement and confirmation of hyperglycemia in diabetic rats. Animals with a blood glucose level of less than 200 mg/dL were excluded.

### Experimental protocol to induce the ulcers

For the induction of ulcers, the animals were anesthetized with intraperitoneal ketamine (80 mg/kg) and xilazin (10 mg/kg). Antisepsis was performed with an oral solution of 0.12% chlorhexidine gluconate in cotton pellets. The ulceration in the left buccal mucosa was performed by abrasion with a number 15 scalpel blade, and a marker with an 8 mm diameter was used for standardization of the lesion area. The surgical technique was standardized for all animals and performed by the same operator^9^.

### Groups and treatment

The animals were randomly divided into five groups by lot:

- Groups with Saline Treatment: Negative Control Group (normoglycemic rats) and a Positive Control Group (diabetic rats);

- Groups with Chamomile Treatment: Chamomile Normoglycemic Group (normoglycemic rats treated with chamomile) and Chamomile Diabetic Group (diabetic rats treated with chamomile);

- Groups with Triamcinolone Treatment: Triamcinolone Group (diabetic rats treated with Triamcinolone).

The five groups were treated every twelve hours for five and ten days (20 treatments in total) with topical application of sterile saline solution in the Negative Control Group and Positive Control Group, Omcilon-A, orabase^®^, 1 mg/g (B-MS, São Paulo, SP, Brazil) in the Triamcinolone Group, or Ad-Muc^®^ 10% ointment (BIOLAB, São Paulo, SP, Brazil) in the Chamomile Group (diabetic and normoglycemic). The application of the drugs was performed with an individual sterile and disposable *microbrush* (KG Sorensen^®^, Cotia, SP, Brazil).

The sacrifice was performed by injection of a lethal dose of 2 mL of 10% chloral hydrate at five and ten days after ulcer induction.

### Clinical evaluation

The animals were weighed and had the blood collected on day zero and on sacrifice day. On the day of sacrifice their ulcers were measured using a 0.5 mm digital pachymeter (L=larger diameter; m=minor diameter) to calculate the area of the ulcer (Area=π x Larger radius x minor radius). All measurements were performed by the same operator^8^. Weight, blood glucose, and ulcer diameter were evaluated in the 5^th^ and 10^th^ days.

### Histological analysis

**Figure 1:**
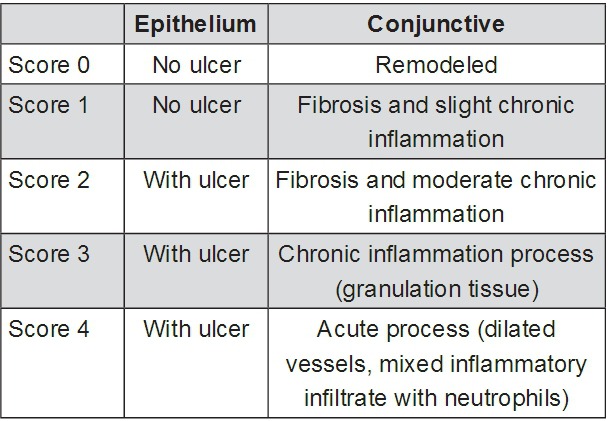
Microscopic analysis of induced oral ulcers (histological scores)

After sacrifice, the collected fragments of buccal mucosa were identified and immersed in 10% neutral formol for 24 hours. After fixation in formol, the specimens were macroscopically analyzed, subjected to dehydration in crescent alcoholic series, diaphanized in xylol, impregnated in paraffin, and melted at 60º C. Then, the fragments were placed in paraffin-forming blocks at room temperature. The fragments were sectioned to a thickness of 5 µm using a microtome (Leica Geosystems, Atlanta, Georgia, USA^®^), and histology was performed using routine coloration by hematoxylin-eosin (HE). The histopathological parameters were determined and scored from 0 to 4 according to previously published criteria (Figure 1)^8^.

### Collagenesis assay

The same fragments were sectioned to a thickness of 3 µm and analyzed by histology using picrosirius staining to evaluate collagen deposition. Tree fields were photographed for each histological slide (200x) (Motic BA310 Microscope with Moticam 2000 2.0 M Pixel USB 2.0 camera attached and software Motic Plus 2.0) (Motic, Medical Diagnostic Systems Co Ltda^®^, Naperville, Illinois, USA). The photomicrographs were quantitatively evaluated using image analysis software (ImageJ^®^, National Institutes of Health, Bethesda, Maryland, USA) after calibrating images by the Color Threshold command (Image>Adjust>Color Threshold) in the RGB function for colors Red (minimum 71, maximum 255), Green (minimum 0, maximum 69) and Blue (minimum 0, maximum 92). After calibration, images were converted to a color scale of 8-bits (Image>Type>8-bit) and binarized (Process> Binary>Make Binary) before the percentage of collagen in a given area, marked in black, was analyzed (Analyze>Analyze Particles). The mean of tree percentages was used as a sample unit^2^.

### Immunohistochemistry assay

After deparaffinization and rehydration, tissue sections (2.5 μm) were also submitted to immunohistochemical assay. Antigenic recuperation was performed by heat in citrate pH 6.0 solution. After cooling, the slides were submitted to peroxidase blocking with H_2_O_2_ 3% solution diluted in PBS (phosphate buffered saline) for 30 minutes.

After protein blocking (PBS) for 1 hour, the specimens were incubated overnight with Tumor Necrosis Factor alpha (TNF-α) (Abcam^®^, Cambridge, UK) in 1:100 dilution. Then, the primary antibody Simple Stain Rat MAX PO (Multi) Universal Immuno-peroxidase Polymer (anti-mouse and -rabbit) (Histofine^®^, Nicherei Biosciences Inc., Tokyo, Japan) was used for 60 minutes. The revelation system 5,5-diaminobenzidine tetrahydrochloride (DAB) (Dako^®^, Carpinteria, CA, USA) was used for 5 minutes and the counter coloration was Harris hematoxylin, which happened for 30 seconds.

The percentage of cells in connective tissue with cytoplasmatic or nuclear expression was grouped into scores: (0) no positive cells; (1 - mild) 1-33% of positive cells; (2 - moderate) 34-66% of positive cells; (3 - intense) 67-100% positive cells. The same score, obtained by two or more observers, was considered as the final score^11^.

### TUNEL assay

Tissue sections (2.5 μm) were also subjected to antigen retrieval with Proteinase K (Dako^®^, Carpinteria, CA, USA) (1:250) for 15 minutes and peroxidase blocking with H_2_O_2_ 3% solution diluted in PBS (phosphate buffered saline) for 5 minutes. After peroxidase blocking, the slides were incubated for 10 seconds with PBS buffer (ready to use) and with Tdt (1:2.5) for 90 minutes at room temperature.

The tissue slides were rinsed with the wash buffer (1:15) for 10 minutes and after this the anti-digoxigenin conjugate (ready to use) was added for 30 minutes. The revelation system was done with DAB (Dako^®^, Carpinteria, CA, USA). The tissue slides were stained with metil green for 10 minutes followed by dehydration, diaphanization, and mounting with coverslips.

The percentage of positive cells in connective tissue and epithelium was grouped into scores: (0) no positive cells; (1 - mild) 1-33% of positive cells; (2 - moderate) 34-66% of positive cells; (3 - intense) 67-100% positive cells. The same score, obtained by two or more observers, was considered as the final score^11^.

### Statistical analysis

The area of ulceration, weight loss, blood glucose change, and percentage of collagen in an area were analyzed by the t-test and ANOVA/Tukey test. The Mann-Whitney test and Kruskall-Wallis/Dunn test were used to histological scores. The relationship between the area of ulceration, weight loss and blood glucose variation in the four experimental groups was analyzed by Pearson correlation. The relationship between these parameters and the histological scores were analyzed by Spearman correlation. All analyses were performed with the software GraphPad Prism 5.0^®^ (GraphPad Software Inc., San Diego, California, USA), with a p<0.05 significance level.

## RESULTS

### Clinical evaluation

**Figure 2:**
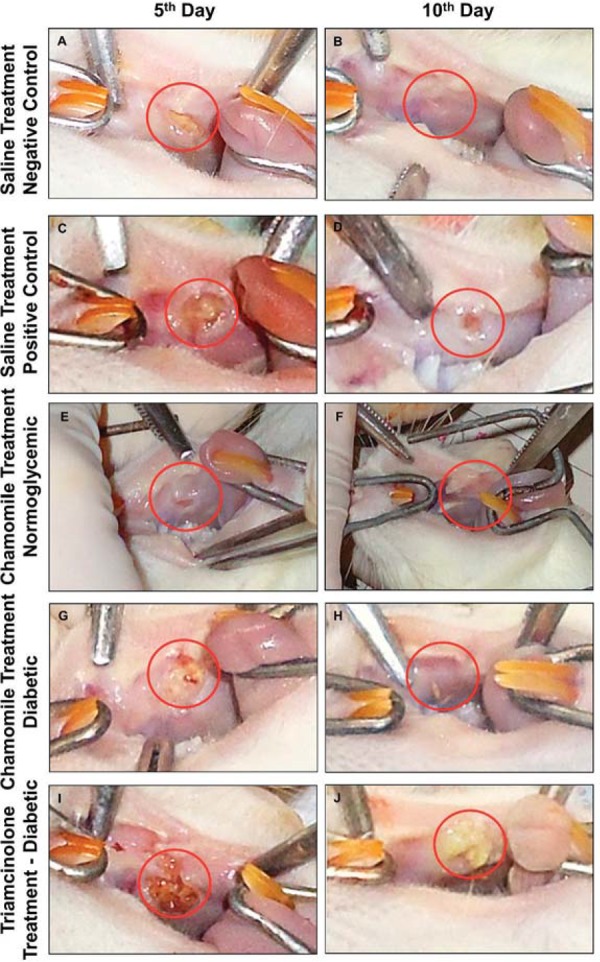
Macroscopic ulcers in the buccal mucosa in the Saline Treatment groups [Negative Control=Normoglycemic Rats (A and B); Positive Control Group=Diabetic Rats (C and D)], Chamomile Treatment Groups [Normoglycemic (E and F) and Diabetic Rats (G and H)], and Triamcinolone Group (I and J), respectively

Dimensional analysis of the ulcer: All experimental groups showed a decrease in ulcer area from the 5^th^ day to the 10^th^ day, except the Triamcinolone Group. On the 10^th^ experimental day, only the group treated with triamcinolone showed a significantly higher ulcer size compared with the Positive Control Group (Figure 2, Table 1).

**Table 1:** Values of the Oral Ulcer Area, Weight Loss, and Blood Glucose Variation on the 5th and 10th experimental days in the Saline Treatment groups (Negative Control=Normoglycemic Rats; Positive Control Group=Diabetic Rats), Chamomile Treatment Groups (Normoglycemic and Diabetic Rats), and Triamcinolone Group

Groups	Clinical Evaluation								
	Area of Ulcer (mm^2^)			Weight Loss (g) [Final weight – Initial weight]			Blood Glucose Changes (mg/dL) [Final Glucose – Initial Glucose]		
	5^th^	10^th^	p1	5^th^	10^th^	p1	5^th^	10^th^	p1
Saline Treatment									
Negative Control (n=13)	14.2±2.2	0.0±0.0	0.003	-25.0±5.4§	-8.3±2.1§	0.0105	-15.4±9.6	7.2±4.5	0.051
Positive Control (n=12)	18.4±2.2	5.5±0.9	0.0005	-19.0±5.1§	-15.0±4.2§	0.561	-134.2±46.0*§	-147.5±22.4*§	0.8183
Chamomile Treatment									
Chamomile Normoglycemic (n=14)	18.9±2.2	0.4±0.2	0.0002	6.2±3.5	19.7±2.9	<0.0001	-16.9±12.1	5.2±5.0	0.1201
Chamomile Diabetic (n=17)	18.5±2.0	4.0±1.3	0.0004	-25.6±1.3§	-21.0±4.6§	0.2477	119.7±13.3†§	10.0±12.6†§	0.0002
Triamcinolone Treatment									
Triamcinolone Diabetic (n=17)	18.8±0.5	10.5±3.3†	0.082	-47.8±3.0†§	-52.1±4.5*§	0.4165	55.8±70.5†§	32.5±24.7†§	0.5206

P1 t-test; Data are shown as the mean ± SE; *p<0.05 compared with the Negative Control Group on the same day; †p<0.05 compared with the Positive Control Group on the same day; ‡p<0.05 compared with the Chamomile Diabetic Group on the same day; §p<0.05 compared with the Chamomile Normoglycemic Group on the same day

Weight loss: After the treatment, Negative Control, Positive Control, Chamomile Diabetic, and Triamcinolone Groups had weight loss relative to day zero. In Negative Control Group and Chamomile Normoglycemic Group, there was a significant recovery of weight from the 5^th^ day to the 10^th^ day. The diabetic groups showed no recovery of weight. The Triamcinolone Group showed significant weight loss compared with the Positive Control Group on the 5^th^ and 10^th^ days of the experiment. The weight gain was significantly higher in Chamomile Normoglycemic group from the 5^th^ day to the 10^th^ day (Table 1).

Blood glucose variation: On the 5^th^ and 10^th^ days of the experiment, the reduction of blood glucose in the Positive Control Group (-134.2±46.0 and -147.5±22.4, respectively) was significantly greater than the Negative Control Group (-15.4±9.6 and 7.2±4.5, respectively) and the Chamomile Normoglycemig Group (-19.6±12.1 and 5.2±5.0, respectively). The Chamomile Diabetic Group (119.7±13.3 on the 5^th^ day and 10.0±12.6 on the 10^th^ day) and the Triamcinolone Group (55.8±70.5 on the 5^th^ day and 32.5±24.7 on the 10^th^ day) showed a significant increase in blood glucose variation compared with the Positive Control Group on both days.

From the 5^th^ day (119.7±13.3) to the 10^th^ day (10.0±12.6), there was a significant reduction of blood glucose variation in the Chamomile Group only (Table 1).

### Histological analysis

Histological analysis: On the 5^th^ day of treatment, Negative Control Group, Positive Control Group, and Triamcinolone Group presented a median score of 4, characterized by the presence of an ulcer associated with acute inflammation. The groups treated with chamomile extract (normoglycemic and diabetic) presented a median score of 3, with persistence of the ulcer and mixed inflammatory infiltrate (mononuclear cells and neutrophilic granulocytes) in the underlying connective tissue. On the 10^th^ experimental day, the Negative Control Group and the Chamomile Diabetic Group presented a median score of 1, represented by the absence of an ulcer, fibrosis and slight chronic inflammation. In the Positive Control Group, we assigned a median score of 2 because there was a verifiable epithelial ulceration, but the underlying connective tissue had moderate fibrosis and chronic inflammation. The Triamcinolone Group had a median score of 4, characterized by the presence of an ulcer and intense acute inflammatory process. Only Chamomile Normoglycemic Group shows epithelium totally remodeled and no inflammation in connective tissue (score of 0).

**Figure 3:**
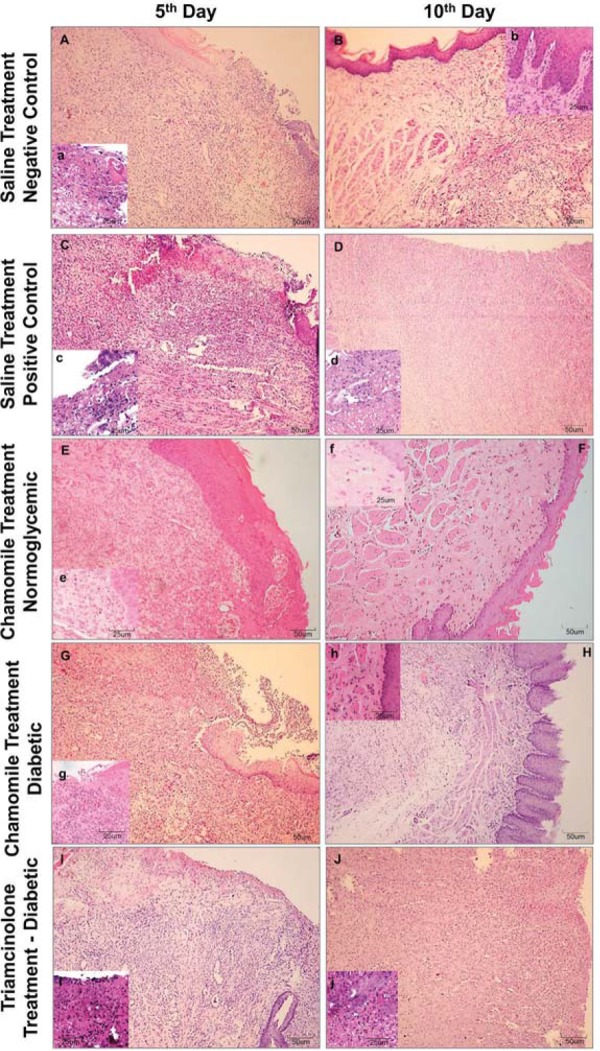
Photomicrograph of ulceration in the buccal mucosa in the Saline Treatment groups [Negative Control=Normoglycemic Rats (A and B); Positive Control Group=Diabetic Rats (C and D)], Chamomile Treatment Groups [Normoglycemic (E and F) and Diabetic Rats (G and H)], and Triamcinolone Group (I and J), respectively. (Hematoxylin & eosin, Capital letters=100x; Small letters=200x)

After statistical analysis, we found a significant reduction of the histological scores of the ulcers from the 5^th^ day to the 10^th^ day in all experimental groups, except in the Triamcinolone Group. On the 5^th^ day, there was no significant difference in the histological scores for the five experimental groups (Figure 3, Table 1). On the 10^th^ day, the Positive Control Group showed histological scores (median=2) significantly higher than the Negative Control Group (median=1) and Chamomile Normoglycemic group (median=0). The Positive Control Group (median=2) and the Chamomile Group (median=1) did not differ significantly; however, the Triamcinolone Group (median=4) showed histological scores that were significantly higher than the Positive Control Group (Table 2).

**Table 2:** Histological Scores and Percentage of Collagen Deposition Area on the 5th and 10th experimental days in the Saline Treatment groups (Negative Control = Normoglycemic Rats; Positive Control Group = Diabetic Rats), Chamomile Treatment Groups (Normoglycemic and Diabetic Rats) and Triamcinolone Group

Groups	Microscopic Evaluation														
	Histological Scores			Percentage of Collagen Deposition Area (%)			TNF-α Immunostaining			TUNEL connective tissue Staining			TUNEL epithelium Staining		
	5^th^	10^th^	p1	5^th^	10^th^	p2	5^th^	10^th^	p1	5^th^	10^th^	p1	5^th^	10^th^	p1
Saline Treatment															
Negative Control (n=13)	4	1	0.0062	39.2±4.9	57.8±1.8	0.0264	3	1	0.0266	3	1	0.0273	3	3	0.9222
	(2,4)	(0,3)					(2,3)	(0,3)		(3,3)	(0,3)		(1,3)	(1,3)	
Positive Control (n=12)	4	2	0.0285	25.8±2.8*	38.7±4.2*	0.0452	3	3	0.3367	3	3	1.0000	3	3	1.0000
	(4,4)	(2,2)*§					(3,3)	(2,3)		(3,3)	(3,3)*§		(2,3)	(3,3)	
Chamomile Treatment															
Chamomile Normoglycemic (n=14)	3	0	0.0074	30.9±4.3	41.0±3.6	0.0297	1	1	0.1683	3	1	0.0469	3	2	0.1683
	(1,3)	(0,1)					(1,1)	(0,1)		(2,3)	(1,3)		(3,3)	(2,2)	
Chamomile Diabetic (n=17)	3	1	0.0041	45.1±1.2†	51.5±6.2	0.1934	3	2	0.0062	3	3	0.3545	3	2	0.032
	(2,4)	(1,3)					(2,3)	(1,3)		(3,3)	(2,3)		(3,3)	(1,3)	
Triamcinolone Treatment															
Triamcinolone (n=17)	4	4	0.5127	38.2±1.9	30.6±4.4‡	0.3251	3	3	1.0000	3	3	1.0000	3	3	0.3545
	(4,4)	(3,4)†§					(3,3)	(3,3)*‡§		(3,3)	(3,3)*§		(2,3)	(3,3)‡§	

p1 Mann-Whitney; The scores are showed as the median (minimum, maximum); p2 t-test. Quantitative data are showed as the mean ± SE; *p<0.05 compared with the Negative Control Group on the same day; †p<0.05 compared with the Positive Control Group on the same day; ‡p<0.05 compared with the Chamomile Diabetic Group on the same day; §p<0.05 compared with the Chamomile Normoglycemic Group on the same day

### Collagenesis

**Figure 4:**
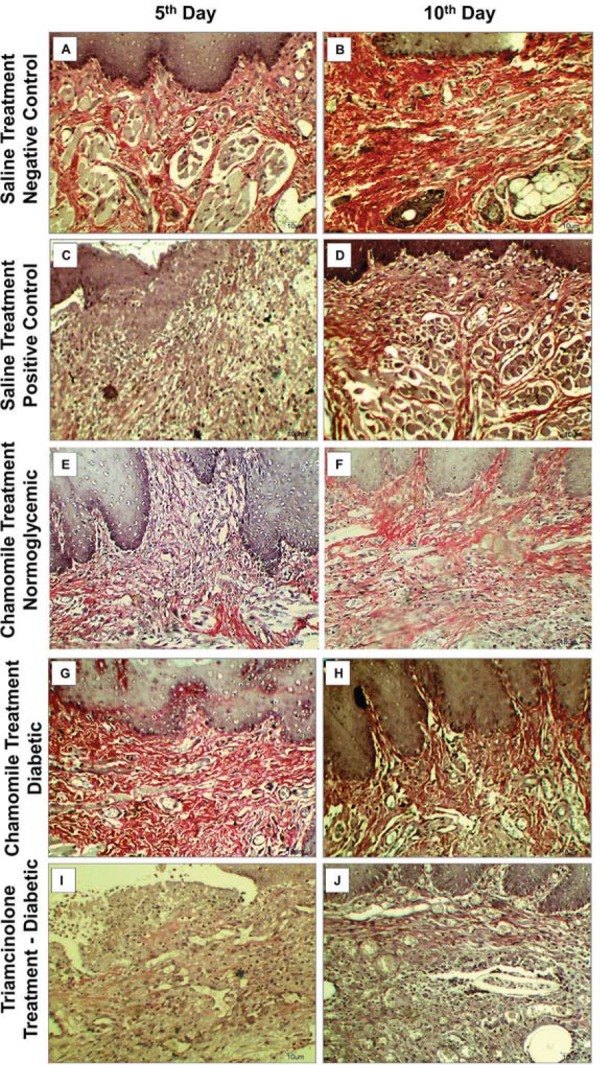
Photomicrograph of ulceration in the buccal mucosa with picrosirius staining in Saline Treatment groups [Negative Control=Normoglycemic Rats (A and B); Positive Control Group=Diabetic Rats (C and D)], Chamomile Treatment Groups [Normoglycemic (E and F) and Diabetic Rats (G and H)], and Triamcinolone Group (I and J), respectively (Picrosirius, 200x)

On the 5^th^ experimental day, the collagen deposition was significantly reduced in the Positive Control Group (25.8±2.8) compared with the Negative Control Group (39.2±4.9). The Chamomile Diabetic Group (45.1±1.2) showed a significantly higher collagen deposition than the Positive Control Group. On the 10^th^ day, the Positive Control Group (38.7±4.2) also had collagen deposition significantly lower than the Negative Control Group (57.8±1.8). Positive Control Group (38.7±4.2), Chamomile Diabetic Group (51.5±6.2), and Chamomile Normoglycemic Group (41.0±3.6) did not differ statistically, but the Chamomile Diabetic Group showed a collagen deposition significantly higher than the Triamcinolone Group (30.6±4.4). Only Negative Control Group, Positive Control Group, and Chamomile Normoglycemic Group showed augment in collagenesis from the 5^th^ to the 10^th^ day (Figure 4, Table 2).

### TNF-α immunostaining

**Figure 5:**
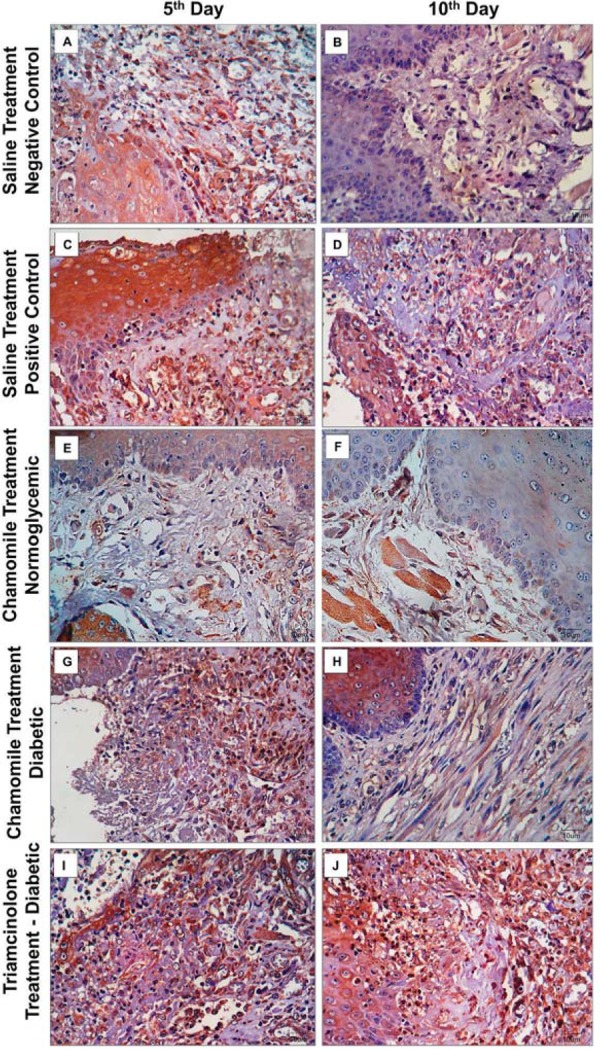
Photomicrograph of ulceration in the buccal mucosa with TNF-α staining Saline Treatment groups [Negative Control=Normoglycemic Rats (A and B); Positive Control Group=Diabetic Rats (C and D)], Chamomile Treatment Groups [Normoglycemic (E and F) and Diabetic Rats (G and H)], and Triamcinolone Group (I and J), respectively (Immunohistochemistry, 400x)

In connective tissue, the immunostaining for TNF-α was augmented on the 5^th^ day. All groups showed a high score (median=3) of TNF-α expression by elevated number of connective tissue cells expressing TNF-α in cytoplasm, except Chamomile Normoglycemic Group (median=1). On the 10^th^ day the Negative Control Group (median=1) and the Chamomile Diabetic Group (median=2) showed significant reduction of TNF-α scores (p=0.0266 and p=0.0062, respectively). Positive Control Group (median=3, p=0.3367), Chamomile Normoglycemic Group (p=0.1683), and Triamcinolone Group (median=3, p=1.0000) did not showed similar reduction, exhibiting a high number of TNF-α positive connective tissue cells. The score of the Triamcinolone Group on the 10^th^ day was statistically higher than chamomile groups (Figure 5, Table 3).

**Table 3:** Correlation between clinical and histological parameters

	Correlation	Negative Control (n=13)	Positive Control (n=12)	Chamomile (n=17)	Triamcinolone (n=17)
Pearson Correlation	Area of Ulcer x Weight Loss	p=0.002* r=–0.766	p=0.010* r=–0.763	p=0.994 r=0.002	p=0.120 r=–0.392
	Area of Ulcer x Blood Glucose Changes	p=0.204 r=0.503	p=0.066 r=–0.573	p=0.089 r=0.471	p=0.999 r=0.000
	Blood Glucose Changes x Weight Loss	p=0.270 r=0.372	p=0.011* r=0.728	p=0.641 r=0.126	p=0.305 r=0.234
Spearman Correlation	Histologic Scores x Area of Ulcer	p<0.001* r=0.836	p=0.046* r=0.640	p=0.011* r=0.601	p=0.020* r=0.573
	Histologic Scores x Weight Loss	p=0.335 r=–0.291	p=0.192 r=–0.425	p=0.266 r=–0.286	p=0.540 r=–0.160
	Histologic Scores x Blood Glucose Changes	p=0.970 r=0.011	p=1.000 r = 0.000	p=0.070 r=0.790	p=0.270 r=–0.284

*p< 0.05, Pearson or Spearman Correlation. Data are shown as p-values and correlation coefficients (r).

### Apoptosis assay

**Figure 6:**
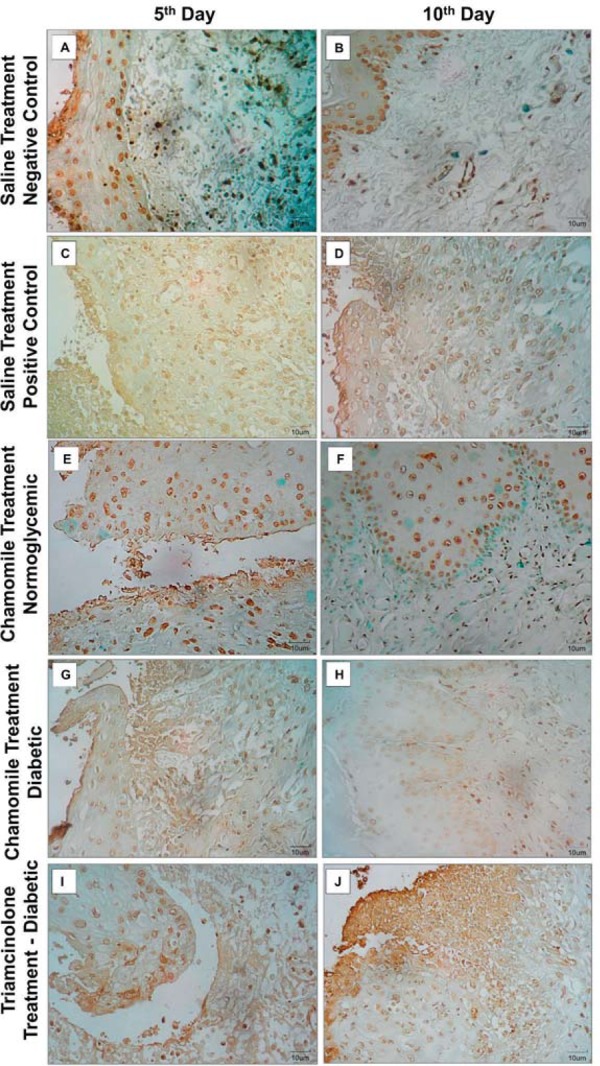
Photomicrograph of ulceration in the buccal mucosa with TUNEL assay in Saline Treatment groups [Negative Control=Normoglycemic Rats (A and B); Positive Control Group=Diabetic Rats (C and D)], Chamomile Treatment Groups [Normoglycemic (E and F) and Diabetic Rats (G and H)], and Triamcinolone Group (I and J), respectively (TUNEL assay, 400x)

Connective tissue: In connective tissue, the number of TUNEL positive cells was elevated. All groups showed a high number of cells in apoptosis (median=3). Only Negative Control Group (p=0.0273) and Chamomile Normoglycemic Group (p=0.0469) showed reduction in score of TUNEL staining on the 10^th^ day (median=1). On the 10^th^ day all animals of Positive Control Group and Triamcinolone Group exhibited more than 66% of TUNEL positive cells (median=3, minimum=3, maximum=3) with statistical difference between Negative Control Group. Chamomile groups did not showed difference in scores of TUNEL positive cells in relation to Negative Control Group (Figure 6).

Epithelium: Similarly, all groups exhibited more than 66% of TUNEL positive epithelial cells on the 5^th^ day. On the 10^th^ day, only Chamomile Normoglycemic Group showed reduction in score of TUNEL positive epithelial cells (median=2, p=0.0320), but there is no differences regarding Positive Control Group (median=3) and Negative Control Group (median=3). The Triamcinolone Group score (median=3) was higher than Chamomile Normoglycemic and diabetic groups on the 10^th^ day (Figure 6).

### Correlation analyses

There was a significant correlation between the area of the oral ulcer and weight variation in the Negative Control Group (p=0.002, r=-0.766) and the Positive Control Group (p=0.010, r=-0.763) because the weight loss was proportional to the area of the ulcer. In other groups, the correlation was not statistically significant (Table 4).

In the Positive Control Group (p=0.011, r=0.728), the correlation between weight variation and blood glucose variation was significant, with weight loss proportional to glucose lowering.

Histological scores presented significant correlation directly with the area of the oral ulcer in all five experimental groups; however, they did not influence the weight or the blood glucose variation (Table 3).

## DISCUSSION

High levels of blood glucose affect oral wound healing negatively. Wound healing in an uncontrolled diabetic patient occurs more slowly compared with normoglycemic or controlled diabetic patients^13^. This delay in oral wound healing can cause chronicity of oral lesions in patients with DM^18^.

In this study, a wound healing deficit was observed in diabetic rats. On the 10^th^ experimental day, we observed characteristics compatible with tissue healing in the normoglycemic group (score=1), but the diabetic group showed persistence of the ulcer and chronic inflammation of the underlying connective tissue (score=2). Regarding fibrogenesis, there was a reduction in collagen deposition in the diabetic rats when compared with the normoglycemic rats at both time points. Reinforcing the histopathology analysis, the macroscopic evaluation showed that on the 10^th^ experimental day the oral ulcers were completely healed in the normoglycemic rats, but not in the diabetic animals.

Animals treated with chamomile extract showed modification of the chronic inflammatory profile on the 5^th^ experimental day, unlike Negative Control, Positive Control, and Triamcinolone Groups. Additionally, on the 10^th^ day, the histological findings for the Chamomile Group were similar to those for the Negative Control Group (normoglycemic), showing total epithelial healing and mild chronic inflammation. In the fibrogenesis evaluation, collagen deposition in the Chamomile Group was significantly higher than in the Positive Control Group (diabetic) and did not differ from the Negative Control Group (normoglycemic).

The anti-inflammatory effects of chamomile have been linked to compounds present in its extract such as flavonoids, alpha-bisabolol, and acetylated derivatives^21^ ^,^ ^26^ ^,^ ^27^. The mechanism of action of chamomile in the inflammatory process involves direct inhibition of ciclooxigenase-2 and synthesis of inflammatory mediators such as prostaglandin E2^28^. In this study, the chamomile group (DCG) was the single treatment that showed reduction in TNF-α expression in diabetic rats that are likely to augment in proinflammatory cytokines corroborating the anti-inflammatory effects of this extract. These actions lead to a reduction in vascular and cellular events, including acute inflammation. Furthermore, alpha-bisabolol has been associated with promoter activity in the formation of granulation tissue during the process of wound repair^4^.

Additionally, the reduction of apoptosis scores only in connective tissue of NCG may be associated with high sensibility of wound fibroblasts. Wound fibroblasts are highly sensitive to oxidative stress that is proportional to TNF-α activation^29^. NCG showed high reduction in TNF scores in connective tissue and have a potent antioxidant activity, leading to reduction of apoptosis only in connective tissue.

The increase in TNF-α and oxidative stress due to elevated levels of glucose decreases cell viability and survival of various cell types, including fibroblasts^17^ ^,^ ^30^. However, there was no improvement in apoptosis profile connective tissue cells in any treatment. Only Negative Control Group had reduction in apoptosis in connective tissue. The reduction in TNF-α expression occurred concomitantly with decreased epithelial cells apoptosis in CDG showing that it can modulate apoptosis of these cells when topically used.

In a study with diabetic rats, oral administration of chamomile extract showed anti-hyperglycemic effects and was able to ameliorate the complications caused by hyperglycemia associated with DM^9^ ^,^ ^14^. In our study, the animals treated with chamomile showed significant reduction of blood glucose variation from the 5^th^ to the 10^th^ experimental days. However, despite reports of the anti-hyperglycemic effects of systemic chamomile, further researches are needed to evaluate this finding because the chamomile was only topically applied to the oral ulcers.

In contrast, therapy with corticosteroids in traumatic lesions has negative effects on wound healing. Steroids affect the synthesis and maturation of collagen, alter the tensile strength of the wounds, inhibit cell function and proliferation, and decrease the antibacterial and phagocytic actions of some defense cells, resulting in a delay in wound healing^1^ ^,^ ^22^. In this study, the Triamcinolone Group exhibited an oral ulcer area that was significantly higher than those of the Negative Control Group and the NCG on the 10^th^ experimental day. The triamcinolone administered topically delayed the repair process, which caused persistence of the ulcer and acute inflammation on the 5^th^ and 10^th^ experimental days. This effect was not observed in other groups. The Triamcinolone Group exhibited less collagen deposition and higher weight loss than the Positive Control Group (diabetic rats), suggesting that this drug inhibits additional wound healing in the oral cavity in diabetic rats.

In this research, there was a significant increase of blood glucose variation on both the 5^th^ and 10^th^ experimental days in animals treated with chamomile and triamcinolone when compared with the untreated diabetic rats. These data can be associated with the antinociceptive effects of both drugs^10^. Chamomile and triamcinolone can reduce nociception, leading to increased food intake and a consequent augment in blood glucose. The size of the ulcerative process in the oral cavity is proportional to the painful symptomatology, and topical drugs with anesthetic or analgesic effects diminish this relationship^16^. Thus, this model is supported by the analysis of the correlation between the area of the oral ulcer and weight loss, since the weight loss was proportional to the area of the ulcer only in the untreated animals (Positive Control Group and Negative Control Group).

The weight loss occurs due to diabetic anorexia, which is coupled with nociception and consequent dysphagia resulting from oral ulceration^3^. This combination may interfere with feeding and be responsible for the variation in blood glucose and difficulty in weight gain observed in an untreated diabetic animals^15^. The results of the present study showed weight loss in all of the experimental diabetic groups; however, the Triamcinolone Group had significantly greater weight loss than the Positive Control Group. Corticosteroids promote a complex metabolic process that results in malnutrition and catabolism. Consequently, this poor health condition of the animals may contribute to the delayed healing and the increase in weight loss^1^.

The corticosteroids, even administered topically, can induce side effects and negatively influence wound healing. The extract of *Matricaria recutita L*. (chamomile) may be an important tool to optimize the healing process of oral traumatic ulcers in DM patients. Clinical trials are needed to confirm these laboratory findings.

## CONCLUSION

Thus, chamomile extract can optimize the healing of traumatic oral ulcers in diabetic rats through the reduction of apoptosis in the epithelium and TNF-α expression. On the other hand, steroid therapy had negative effects on the healing process of oral ulcers.
